# Long-term reproducibility of GC-IPL thickness measurements using spectral domain optical coherence tomography in eyes with high myopia

**DOI:** 10.1038/s41598-018-29466-8

**Published:** 2018-07-23

**Authors:** Min-Woo Lee, Kee-Sup Park, Hyung-Bin Lim, Young-Joon Jo, Jung-Yeul Kim

**Affiliations:** 1 Department of Ophthalmology, Chungnam National University College of Medicine, Daejeon, Republic of Korea; 2 Department of Ophthalmology, Armed Forces Capital Hospital, Seongnam, Republic of Korea

## Abstract

Although ganglion cell inner plexiform layer (GC-IPL) analysis in the patients with high myopia is useful, there have been few reports to analyze of the reliability for long-term measured GC-IPL thickness. We aimed to analyze the long-term reproducibility of thickness measurements of the GC-IPL using spectral-domain optical coherence tomography (SD-OCT) in patients with high myopia and identify factors that affect such reproducibility. 99 eyes from 99 patients with high myopia without any other ophthalmc disorder such as glaucoma or retinal diseases were included. Two serial SD-OCT (Cirrus-HD) macular scans taken at ≧1 year intervals were analyzed. The intraclass correlation coefficient (ICC), coefficient of variation (CV), and test-retest standard deviation (TRTSD) of GC-IPL thickness measurements were assessed. The ICC, CV, and TRTSD of the mean GC-IPL thicknesses were 0.883, 5.9%, and 2.74, respectively. The ICCs of the six-sector GC-IPL thicknesses ranged from 0.740 to 0.904. The CVs of the minimal and all sectoral GC-IPL thicknesses were <10%. Measurement variances for the mean GC-IPL thicknesses showed significant relationshiups with chorioretinal atrophy and posterior staphyloma. There is high long-term reproducibility in GC-IPL thickness measurements using SD-OCT in high-myopia patients. The factors affecting this reproducibility include chorioretinal atrophy and posterior staphyloma.

## Introduction

High myopia is one of the most common causes of visual loss, and is especially prevalent in Asia. The prevalence of myopia has been reported to be as high as 80% in Asia and 25% in other regions of the world^1–4^. Although most refractive errors can be corrected with glasses or contact lenses, high myopia, which is defined as a spherical equivalent of −6.0 diopters (D) or less, may result in a variety of irreversible retinal or optic nerve disorders. In addition, the Tajimi Study reported that myopic macular degeneration is the most common cause of binocular and monocular blindness in Japan^5^, and Xu *et al*. reported that pathological myopia is the second most common cause of low vision and blindness in Chinese people ≥40 years of age^6^. This is because the fiber diameter is small and histologically immature in a highly myopic eye, so that chorioretinal atrophy, choroidal neovascularization, and macular retinoschisis may develop as the structure of the highly myopic eye continuously expands and is made thinner by fewer cross-linkages when compared with an emmetropic eye^7–9^. Because conformational changes of the retina may contribute to the causes of diverse diseases, it is important to characterize these changes in highly myopic eyes.

The thicknesses of the macula, ganglion cell-inner plexiform layer (GC-IPL), and retinal nerve fiber layer (RNFL) can be quantitatively measured using spectral domain optical coherence tomography (SD-OCT), which is now commonly used and measures various parameters of the optic nerve head. Because SD-OCT has high reproducibility, it is valuable for diagnosing glaucoma and optic nerve and retinal disorders.

There are special problems in diagnosing the many optic nerve disorders in high-myopia patients because of different sizes and shapes of the optic nerve head and a thinner RNFL compared with an emmetropic eye. Characterization of the GC-IPL thickness may greatly contribute to the diagnoses and monitoring of these disorders^10^. We therefore determined the long-term reproducibility of GCIPL thickness measurements by SD-OCT in high-myopia patients, and identified factors that affected the reproducibility.

## Results

### Demographics

A total of 99 eyes were included in the study. The mean age, S.E, BCVA, IOP, and AXL were 45.2 ± 17.5 years, −7.65 ± 6.12 D, 0.12 ± 0.25 (logMAR), 15.7 ± 2.6 mmHg, and 28.30 ± 1.99 mm, respectively. Using OCT, the mean signal strength, GC-IPL thickness, and CMT were 6.5 ± 1.1, 72.9 ± 12.2 μm, and 258.6 ± 9.0 μm, respectively. Chorioretinal atrophy was detected in 47 eyes (47.5%) and posterior staphyloma in 27 eyes (27.3%) (Table 1). The initial measurement in 46 eyes showed high repeatability (Table 2).

**Table 1:** Demographics and baseline characteristics.

Age (years, mean ± SD)	45.2 ± 17.5
Male gender (%)	52 (52.5%)
Right laterality (%)	47 (47.5%)
Phakic eye (%)	72 (72.7%)
BCVA (logMAR, mean ± SD)	0.12 ± 0.25
Spherical equivalent (D, mean ± SD)	−7.65 ± 6.12
IOP (mmHg, mean ± SD)	15.7 ± 2.6
Keratometry (D, mean ± SD)	43.3 ± 2.2
Axial length (mm, mean ± SD)	28.30 ± 1.99
Mean interval duration (month, mean ± SD)	27.4 ± 16.8
Mean signal strength (mean ± SD)	6.5 ± 1.1
Mean GC-IPL thickness (μm, mean ± SD)	72.9 ± 12.2
Mean CMT (μm, mean ± SD)	258.6 ± 9.0
Chorioretinal atrophy (%)	47 (47.5%)
Posterior staphyloma (%)	27 (27.3%)

SD, standard deviation; logMAR, logarithm of the minimum angle of resolution; BCVA, best-corrected visual acuity; D, diopters; IOP, intraocular pressure; GC-IPL, ganglion cell-inner plexiform layer; CMT, central macular thickness.

**Table 2:** Repeatability of initial measurement of ganglion cell-inner plexiform layer thicknesses in high-myopia patients.

	ICC	COV (%)	TRTSD
Mean thickness	0.972	2.9	1.36
Minimal thickness	0.869	9.5	2.97
Sectoral thickness			
Superior	0.965	2.9	1.44
Superotemporal	0.912	5.3	2.16
Inferotemporal	0.882	5.0	2.26
Inferior	0.730	8.9	3.42
Inferonasal	0.789	5.0	2.72
Superonasal	0.851	5.1	2.39

ICC, intraclass correlation coefficient; COV, coefficient of variation; TRTSD, test-retest standard deviation.

### Long-term reproducibility of GC-IPL thickness parameters

Regarding the long-term reproducibility of GC-IPL thickness measurements by SD-OCT in high-myopia patients, the ICC of the mean GC-IPL thickness was high (0.883), and those of the six-sector GC-IPL thicknesses ranged from 0.740 (superotemporal) to 0.904 (superonasal). In addition, the COVs of the mean and all sectoral GC-IPL thicknesses were <10%, indicating high reproducibility (Table 3).

**Table 3:** Long-term reproducibility of ganglion cell-inner plexiform layer thickness parameters in high-myopia patients.

	ICC	COV (%)	TRTSD
Mean thickness	0.883	5.9	2.74
Minimal thickness	0.873	15.1	4.61
Sectoral thickness			
Superior	0.837	9.6	4.34
Superotemporal	0.740	9.5	4.25
Inferotemporal	0.859	6.7	3.36
Inferior	0.793	9.4	4.16
Inferonasal	0.820	8.8	4.02
Superonasal	0.904	7.9	3.80

ICC, intraclass correlation coefficient; COV, coefficient of variation; TRTSD, test-retest standard deviation.

### Factors affecting the reproducibility of GC-IPL thickness measurements in high-myopia patients

**Figure 1:**
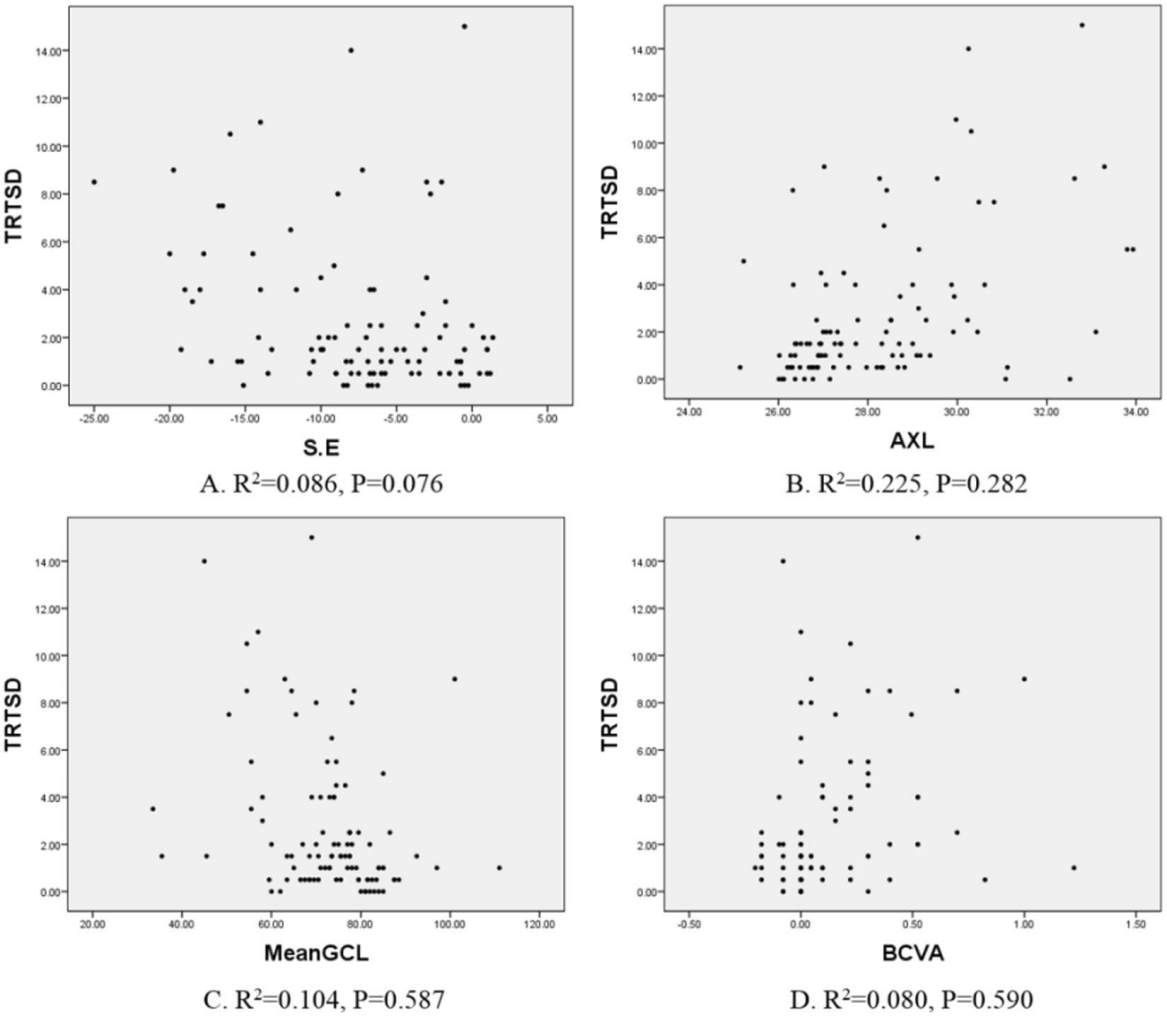
Scatterplots showing the correlations between biometrics, such as (**A**) spherical equivalent (S.E.), (**B**) axial length (AXL), (**C**) ganglion cell layer thickness, (**D**) best-corrected visual acuity (BCVA), and test-retest standard deviation (TRTSD). The correlation coefficient (R^2^) determined by linear regression analyses was used to determine the strength of correlations between variables. Factors determined statistically significant using univariate linear regression analyses, were not statistically significant using multivariate linear regression analyses.

**Figure 2:**
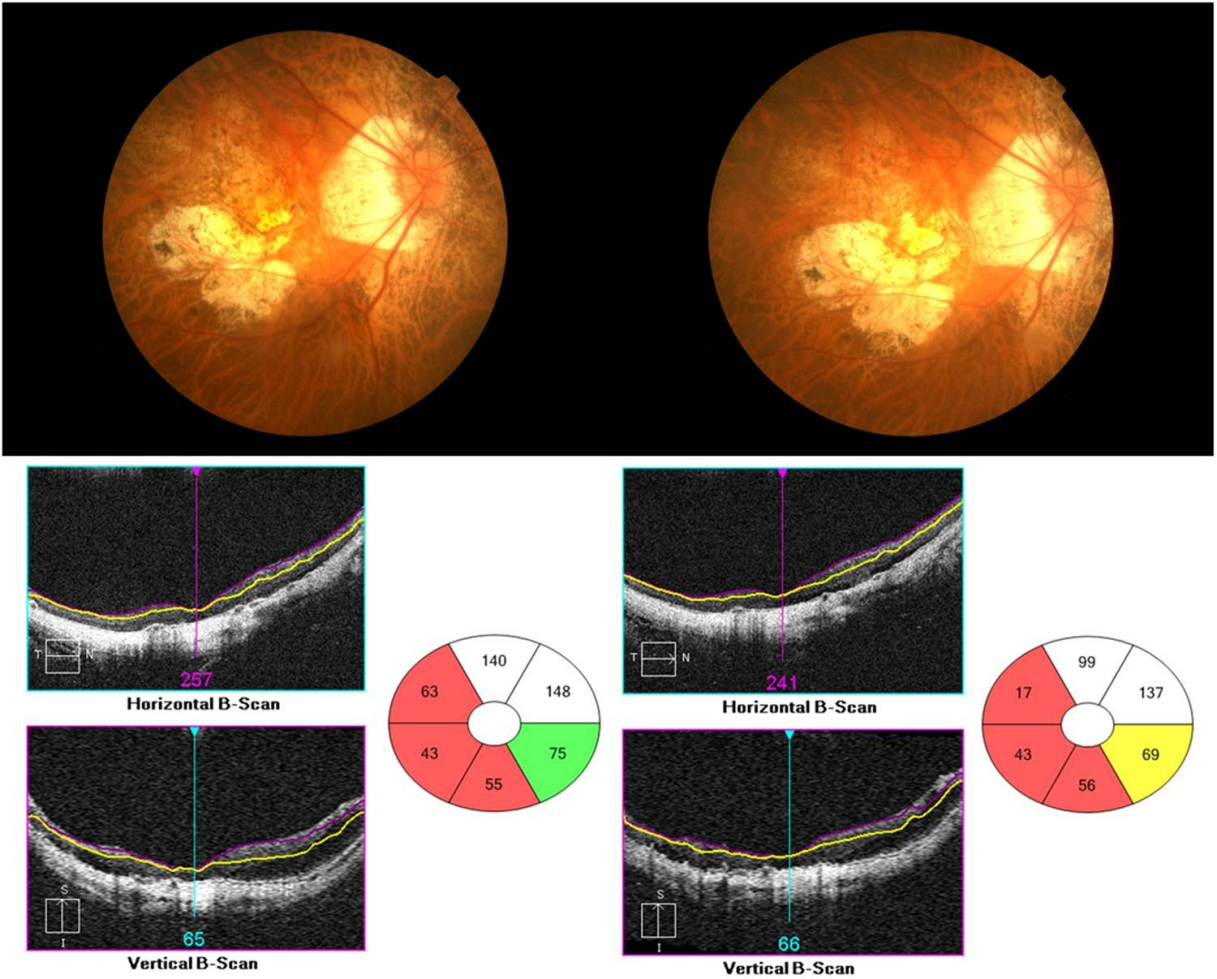
Fundus photographs and ganglion cell-inner plexiform layer (GC-IPL) measurements involving a significance map and horizontal B-scans using spectral domain optical coherence tomography. This patient had extensive chorioretinal atrophy at the first visit (left), and after 2 years (right), the area of chorioretinal atrophy was slightly larger and the GC-IPL thickness was thinner (right). The shape of the yellow segmentation line was also altered to an irregular shape.

To identify factors that affected the reproducibility of GC-IPL thickness measurements, univariate and multivariate linear regression analyses were performed for the TRTSD of the mean GC-IPL thickness (Table 4). Univariate analyses showed that the S.E (B, −0.154; P = 0.003), BCVA (B, 3.723, P = 0.003), AXL (B, 0.765; P < 0.001), chorioretinal atrophy (B, 2.658; P < 0.001), posterior staphyloma (B, 3.512; P < 0.001), and mean GCIPL thickness (B, −0.83; P = 0.001) were significant factors that affected the reproducibility (Fig. 1). Multivariate analyses of these factors showed that there were significant results for chorioretinal atrophy (B, 1.746; P = 0.005) and posterior staphyloma (B, 2.149; P = 0.005) (Fig. 2). In patients with chorioretinal atrophy and posterior staphyloma, the ICC of the mean GC-IPL thickness was 0.583 and 0.621, respectively, and COV was 9.2 and 12.4, respectively (Tables 5 and 6); they showed relatively low reproducibility.

**Table 4:** Univariate and multivariate linear regression analyses of correlations between clinical and anatomical parameters and test-retest standard deviations.

	Univariate		Multivariate	
	B (95% CI)	P value	B (95% CI)	P value
Age	0.025 (−0.011 to 0.061)	0.165	—	—
Sex	0.268 (−0.997 to 1.533)	0.676	—	—
Laterality	−0.318 (−1.582 to 0.946)	0.619	—	—
Lens	0.507 (−0.909 to 1.923)	0.479	—	—
f/u interval	0.016 (−0.022 to 0.054)	0.406	—	—
S.E.	−0.154 (−0.255 to −0.053)	**0.003**	−0.088 (−0.186 to 0.010)	0.076
BCVA	3.723 (1.327 to 6.120)	**0.003**	0.660 (−1.763 to 3.083)	0.541
IOP	0.041 (−0.201 to 0.283)	0.737	—	—
Keratometry	0.276 (−0.004 to 0.557)	0.054	—	—
Axial length	0.765 (0.479 to 1.051)	**<0.001**	0.235 (−0.196 to 0.666)	0.282
Chorioretinal atrophy	2.658 (1.510 to 3.805)	**<0.001**	1.746 (0.532 to 2.960)	**0.005**
Posterior staphyloma	3.512 (2.281 to 4.742)	**<0.001**	2.149 (0.673 to 3.624)	**0.005**
Mean GC-IPL thickness	−0.083 (−0.132 to −0.034)	**0.001**	−0.014 (−0.064 to 0.036)	0.587
Mean signal strength	−0.507 (−1.092 to 0.079)	0.089	—	—
Mean CMT	0.010 (−0.010 to 0.029)	0.340	—	—

CI, confidence interval; f/u, follow up; S.E., spherical equivalent; BCVA, best-corrected visual acuity; IOP, intraocular pressure; GC-IPL, ganglion cell-inner plexiform layer; CMT: central macular thickness.

Values with P < 0.05 are shown in bold.

**Table 5:** Long-term reproducibility of ganglion cell-inner plexiform layer thickness parameters in high-myopia patients with chorioretinal atrophy.

	ICC	COV (%)	TRTSD
Mean thickness	0.583	9.2	4.14
Minimal thickness	0.810	19.8	5.00
Sectoral thickness			
Superior	0.735	16.0	7.05
Superotemporal	0.502	15.6	6.96
Inferotemporal	0.549	10.6	5.35
Inferior	0.696	12.0	5.10
Inferonasal	0.681	13.2	5.83
Superonasal	0.717	12.7	5.83

ICC, intraclass correlation coefficient; COV, coefficient of variation; TRTSD, test-retest standard deviation.

**Table 6:** Long-term reproducibility of ganglion cell-inner plexiform layer thickness parameters in high-myopia patients with posterior staphyloma.

	ICC	COV (%)	TRTSD
Mean thickness	0.621	12.4	5.30
Minimal thickness	0.664	19.2	5.22
Sectoral thickness			
Superior	0.708	17.9	7.54
Superotemporal	0.590	19.5	8.22
Inferotemporal	0.766	14.5	7.26
Inferior	0.472	16.7	6.98
Inferonasal	0.650	15.4	6.20
Superonasal	0.849	15.8	6.87

ICC, intraclass correlation coefficient; COV, coefficient of variation; TRTSD, test-retest standard deviation.

## Discussion

High myopia, a major cause of irreversible vision loss, is especially prevalent in Asia. Saw *et al*. stated that highly myopic patients without other ophthalmic diseases should not be overlooked because a more severe form of myopia can lead to a greater risk of glaucoma, retinal detachment, chorioretinal atrophy, and lacquer cracks^11^. Numerous studies have already shown that high myopia causes diverse conformational changes of the eye. Using SD-OCT, Lam *et al*. reported that the fovea could be flattened in high-myopia patients, which is likely to be a presymptom of vitreoretinal traction^12^. Natsuko *et al*. reported that AXL continually increased, even in high-myopia patients ≥45 years of age with posterior staphyloma, although in general, AXL developed until the age of 13 years, and then was maintained throughout adulthood^13^. Visual loss could be caused by choroidal blood vessels and the neuroretina being pulled by physical tension with the growing AXL^13^. Overall, many complications can develop in high-myopia patients because of conformational changes. To reduce and treat them properly at an early stage, such changes in a myopic eye should be carefully observed.

SD-OCT directly images retinal and optic nerve structures with high resolution, and is used to treat and study various ophthalmological diseases by quantitatively measuring a variety of parameters. Rho *et al*. reported that the reproducibility of long-term RNFL thickness measurements over more than 18 months using SD-OCT in glaucoma patients was very high, with ICC, COV, and TRTSD values of 0.994, 4.2%, and 1.46, respectively^14^. Kim *et al*. reported that the long-term reproducibility of GC-IPL thickness measurements using SD-OCT in glaucoma patients were also high, with ICC, COV, and TRTSD values of 0.993, 3.9%, and 5.32, respectively^15^. In a similar manner, Tito *et al*. analyzed the reproducibility of CMT measurements by SD-OCT in patients with diabetic macular edema, and also found high values^16^.

**Figure 3:**
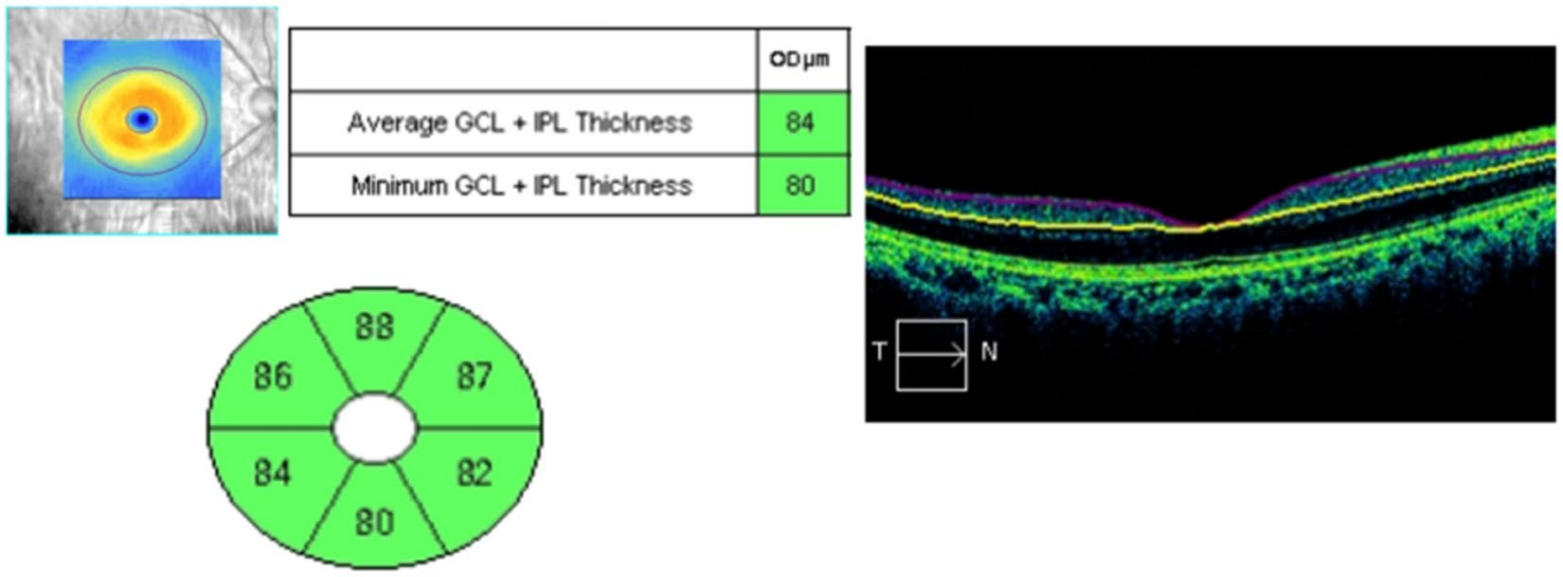
The thickness of the ganglion cell-inner plexiform layer (GC-IPL) using spectral domain optical coherence tomography. The significance map of the GC-IPL shows the average, minimum, and six-sector GC-IPL thicknesses (left). The GC-IPL is auto-segmented and designated using yellow and purple lines (right).

Although SD-OCT reproducibility is required in many diseases, studies of the long-term reproducibility of SD-OCT parameters in high-myopia patients have not yet been reported. This study therefore determined the long-term reproducibility of mean GC-IPL thicknesses using SD-OCT in high-myopia patients, and identified factors affecting this reproducibility. The ICC and COV of the mean GC-IPL thicknesses were 0.883 and 5.9%, respectively, which showed high reproducibility. In addition, those of each sector thickness were ≥0.7 and <10%, respectively, which were also indicative of high reproducibility (Table 3). Univariate linear regression analyses of the TRTSD showed significant results for the S.E, BCVA, AXL, chorioretinal atrophy, and posterior staphyloma, while multivariate linear regression analyses showed significant results for chorioretinal atrophy and posterior staphyloma. It is believed that this is because the measurements were performed in an auto-algorithm of SD-OCT by predicting retina thickness incorrectly owing to conformational abnormalities of the retina, and it corresponds to the results of the study for the repeatability of GC-IPL thickness measurement in a variety of retinal diseases conducted at this institution^17^. Moreover, as shown in Fig. 3, the thickness could be altered by long-term changes of the lesion itself in cases of chorioretinal atrophy or posterior staphyloma. We predicted that the BCVA or AXL would affect the reproducibility, but we found no significant results in multivariate linear regression analyses. However, Fig. 2 indicates that they showed significant results in univariate analyses; TRTSD tended to increase depending on the AXL, so the AXL could not be excluded as a factor affecting reproducibility. Nonetheless, it is thought that the AXL exerts less influence on reproducibility than posterior staphyloma or chorioretinal atrophy, which directly causes conformational abnormalities of the eye.

Chorioretinal atrophy, a type of chorioretinal degeneration with a relatively well-defined boundary of the posterior pole, was observed in 47 eyes (47.5%) in this study. It was also found in 19.3% of the eyes in a Singapore study and 60.9% of the eyes in a Japanese study of high-myopia patients^18,19^. Chorioretinal atrophy is characterized by a lower spherical equivalent, and a high incidence as the AXL becomes longer^18^. The incidence and prevalence varies among studies because the average refractive error and AXL also vary among the different groups in these studies. Tae *et al*. reported that cases with size-limited temporal peripapillary atrophy detected in childhood penetrated the entire posterior pole in adulthood; this study thus emphasized the importance of juvenile chorioretinal atrophy^20^. Liu *et al*. reported that high myopia was more likely to pathologically progress in eyes with chorioretinal atrophy^21^. In addition, Ohno-Matsui *et al*. reported that the area of chorioretinal atrophy by swept-source OCT showed that the outer retina, the RPE, and the choroid were absent, and the inner retina appeared to be attached directly to the sclera^22^. Although inner retina is more intact than outer retina, segmentation error could occur because of too thinned inner retina to separate each layer, or crushed separation resulting in unity of RNFL and GC-IPL on OCT images. So it should be noted that chorioretinal atrophy may affect GC-IPL thickness measurements using SD-OCT if found in high-myopia patients, and also should be noted that it can increase the prevalence of myopia-related complications.

Posterior staphyloma is characterized by a protrusion of the posterior shell of the eye, and causes chorioretinal atrophy and choroidal neovascularization. The present study found posterior staphyloma in 27 eyes (27.3%); however, prevalence varies considerably among previous studies due to inconsistent definitions for myopia. Huang *et al*. reported that both the prevalence and depth of posterior staphyloma were more elevated and intensified, respectively, with increasing age, and the shape of posterior staphyloma was also altered. They also reported that the conformational changes of posterior staphyloma played a more important role in the progression of retinal degeneration progression than the AXL^23^. In the present study, although not classified by shape, posterior staphyloma consequently had a more significant effect on the reproducibility of GC-IPL thickness measurements compared with the AXL. The segmentation error seemed to occur when retinal layers, which contour changes abruptly or irregular, are measured in the posterior staphyloma. Thinned inner retina could be another reason for the segmentation error like the chorioretinal atrophy. These points should be considered if posterior staphyloma is detected in patients, and conformational changes should be carefully observed, although AXL is still an important factor.

This study had some limitations. The data were analyzed retrospectively, and the criteria for posterior staphyloma and chorioretinal atrophy were subjective. In addition, there is some possibility that two scans may be insufficient to determine the reproducibility of the measurements, and possibility of low reliability of some measurements due to relatively low signal strength. However, studies of the reproducibility of OCT parameters are now being conducted for diverse diseases, and the long-term reproducibility of GC-IPL thickness measurements in high-myopia cases and the factors affecting these measurements have not been previously reported.

In conclusion, there was high long-term reproducibility in GC-IPL thickness measurements by SD-OCT in high-myopia patients; the factors affecting this reproducibility included chorioretinal atrophy and posterior staphyloma. Based on these results, the analyses of GC-IPL thickness measurements should be carefully conducted in high-myopia patients.

## Methods

### Subjects

This retrospective study involved patients admitted to our hospital from February 2012 to April 2017. This study adhered to the tenets of the Declaration of Helsinki and the study was approved by the institutional review board of the Chungnam National University Hospital. The requirement for obtaining informed patient consent was waived due to the retrospective nature of the study. A detailed history, best-corrected visual acuity (BCVA), intraocular pressure (IOP) using noncontact tonometry, spherical equivalent (S.E), axial length (AXL) using an IOL Master (Carl Zeiss, Jena, Germany), keratometry, SD-OCT and B-scans were performed, and those with a SE of −6.0 D or less or an AXL ≥ 26.0 mm were selected for the study. To identify short-term repeatability of GC-IPL measurement, the measurements were performed twice with 5-minute interval in 46 eyes at initial visit. To analyze long-term reproducibility, the measurements of every patient were performed twice with at least 1-year interval. Patients with macular lesions, such as aged-related macular degeneration; epiretinal membrane; optic neuropathies, such as glaucoma; and those treated with other intraocular surgeries besides cataract were excluded. We determined the chorioretinal atrophy with binocular stereoscopic ophthalmoscopy including grades 2 through 4; a grade 2 designation of chorioretinal atrophy had a total area of geographic atrophy less than or equal to 2 disk areas. The posterior staphyloma with the B-scan images including one or more grades; a grade 1 staphyloma had a contour change of less than or equal to 2 mm. The grades of the lesions were based on the criteria of Steidl *et al*.^24^. The determination of both lesions was made by 2 of the authors (JYK, MWL).

### OCT measurements

The OCT measurements were performed by a skilled examiner with a 512 × 128 macular cube and 200 × 200 optic cube scanning protocol using a Cirrus HD OCT (Carl Zeiss Meditec, Dublin, CA, USA).

The thickness of the GC-IPL was measured using a ganglion cell analysis (GCA) algorithm from the Cirrus HD OCT. The ganglion cell analysis algorithm automatically measured GC-IPL thickness by identifying the outer boundaries of the RNFL and inner plexiform layer of the macula using three-dimensional information from the macular cube. Mean, minimal, and six-sector (i.e., superior, superotemporal, superonasal, inferior, inferotemporal, and inferonasal) GC-IPL thicknesses were measured by the algorithm (Fig. 3), and the central macular thickness (CMT) was measured using a retinal map analysis system. Cases that showed a signal strength <5 after examination were excluded.

### Statistical analysis

To identify the repeatability and reproducibility of GC-IPL thickness measurements of high-myopia patients, the intraclass correlation coefficient (ICC), coefficient of variation (COV), and test-retest standard deviation (TRTSD) of the mean, minimal, and six-sector GC-IPL thicknesses were calculated. ICC is the ratio of the subject variance to the total variance, and means less variability when close to 1 at the same examination. COV (%) was calculated as 100 × SD/overall mean, and values < 10% indicated good reproducibility. TRTSD was calculated as the square root of the within-subject mean square error.

To identify factors related to reproducibility in measuring GC-IPL thicknesses in high-myopia patients, linear regression analyses were conducted for the TRTSD of mean GC-IPL thickness by investigating ocular variables (including demographics, AXL, and keratometry) and the disease status of patients (whether they had chorioretinal atrophy or posterior staphyloma). B values and 95% confidence intervals (CIs) were calculated, and multivariate analyses were performed for significant values of P < 0.05 obtained by univariate analyses.

### Data availability

Data supporting the findings of the current study are available from the corresponding author on reasonable request.
